# Datasets on the challenges of forced displacement and coping strategies among displaced women in selected Internally Displaced Persons׳ (IDPs) camps in Nigeria

**DOI:** 10.1016/j.dib.2018.07.042

**Published:** 2018-07-27

**Authors:** Faith Osasumwen Olanrewaju, Femi Omotoso, Joshua Olaniyi Alabi

**Affiliations:** a Covenant University, Nigeria; b Ekiti State University, Nigeria

**Keywords:** Coping strategy, Displacement, Internally Displaced Persons (IDPs), IDP camps, Women IDPs

## Abstract

The phenomenon of internal displacement has always existed. It however became the subject of significant concern for the international community since after World War 2, with the violation of the human rights of the displaced arising mostly from the intensification of intra-state wars around the world. The article presented an integrated data on the problems of forced displacement and adopted coping strategies among displaced women in selected IDPs camps in Nigeria. The study used a qualitative approach with a descriptive survey to explain the major problems of forced displacement. The population included women and focus group discussion (FGD) guide was adopted to elicit responses from the study population. Data was described with the use of a 3-D Chart and the data-set is broadly available for further investigation. The findings identified lack of adequate care and financial lack as the major challenges affecting displaced women while economic opportunities was the most significant coping strategy. It was recommended that government and intervening humanitarian agencies will consistently adopt reliable legal and institutional framework for the management of internal displacement and displaced victims in Nigeria.

**Specification Table**

**Table:** 

**Subject area**	Conflict Management
**More Specific Subject Area:**	Internal Displacement
**Type of Data**	Primary source of data
**How Data was Acquired**	Through Focus Group Discussion (FGD) guide
**Data format**	Data were described and thematized
**Experimental Factors**	Population comprised selected women IDPs in Nigeria. The use of adapted FGD guide was employed to investigate the problems of forced displacement and coping strategies adopted by displaced women.
**Experimental features**	The scale and complexity of internal displacement in Nigeria means that significant efforts are required to provide an effective, large-scale and well-coordinated humanitarian response.
**Data Source Location**	FDGs were conducted in Yola (in the North-East geo-political zone) and Abuja (in the North Central geo-political zone)
**Data Accessibility**	Data is made available
**Adapted from:**	Horn [6]; Hines and Balletto [5]; Kruger [9]

**Value of data**

• The data can be used by government and managers to properly develop a National displacement policy for Nigeria.
• The data can help humanitarian actors to effectively manage available resources for addressing displacement crisis.
• The data provides ample knowledge on how to develop a framework for the adoption of global best practices on the management of internal displacement in Nigeria. This will reflect the strategies that have worked for the country and what more needs to be done to reduce displacement and support the displaced.
• The datasets provide a critique of the intra-gender need insensitivity of humanitarian actors towards the displaced persons and how this violates the rights of displaced women living in IDP camps in Nigeria. Thus, the data will also expose government and other humanitarian agencies to the need for intra-gender need vulnerabilities evaluation and needs-based targeted interventions.
• Generally, data acquired from this study would be significant in opening-up a new scope for strengthening partnerships and operational responses and promoting stronger political agenda on internal displacement.

## 1 Data

The increase of the instances of forced displacement is a global trend. Forced displacement is not a new phenomenon, although the recent spate of displacement, as well as the evaluation of the policies on internal displacement have become worrisome. While the Guiding Principles on Internal Displacement is the only global document on internal displacement, it is not binding despite the fact that Internally Displaced Persons (IDPs) constitute a larger number than the refugee population. The domestication of the Guiding Principles around the world have not been encouraging, as most developing countries such as Nigeria, generating most of the global displacement figure have not domesticated the document [3], [8], [11]. Internal displacement (whether due to conflict, natural disasters, or large-scale development projects amongst other causes) is often linked to state fragility characterised by weak governance, fragile institutions, corruption and mismanagement of public funds leading to unequal distribution of wealth and political and economic marginalisation of large parts of the population [1]. In light of the critical humanitarian dilemma associated with internal displacement, the aim of this data article is to assess the challenges associated with internal displacement in Nigeria as well as the coping strategies adopted by displaced women.

The study is qualitative in nature and data were retrieved from females of different social marital categories (single girls, married women, widowed and elderly women) in Nigeria. The population of this study included all Boko Haram terrorism displaced women in Nigeria living in the selected IDP camps which is 726. The sample size of this study is 116 respondents. Samples were deduced for FGD sessions. The use of FGD in this study is important because it gives room for in-depth probing that is likely to produce detailed information necessary for implementing intervention programmes in the selected camps which are Malkohi IDP camp, Adamawa state (symbolized with the acronym MIC and referred to as Camp 1), St Theresa’s Catholic Church IDP camp (symbolized with the acronym STCC and referred to as Camp 2) and Durumi IDP settlement, Abuja (symbolized with the acronym AIS and referred to as Camp 3). FGD questions guides were structured and focused on various issues such as the challenges faced by the displaced women in IDP camps and survival or coping mechanisms adopted [6].

FGDs were conducted in Yola (in the North-East geo-political zone) and Abuja (in the North Central geo-political zone). To avoid non-inclusive, twelve (12) focus group discussions were conducted altogether in the three camps (i.e. 4 FGDs in each camp). Each FGD session consisted of eight to ten discussants. At the end of the day, one hundred and sixteen (116) focus group participants were involved in the FGD sessions. Effort was made to ensure that each group was fairly homogenous with respect to the camps, age and marital status in order to minimize inhibitions in the flow of the discussions.

**Fig. 1:**
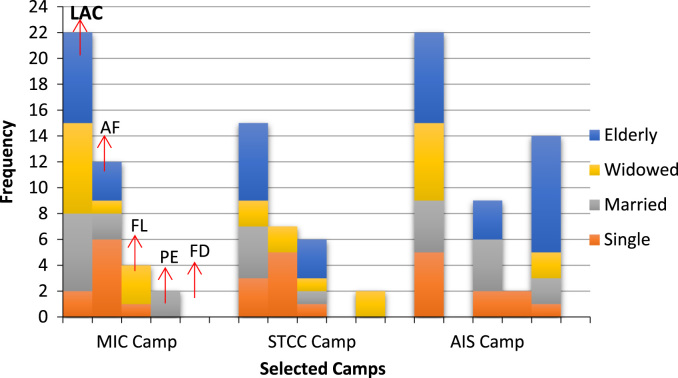
Challenges Internally Displaced Persons faced in selected IDP camps. Keys: Lack of adequate care (LAC); Absence of Freedom (AF); Financial Lack (FL); Poor Education (PE); Family Dis-integration (FD).

Therefore, the graph easily show that the major challenges displaced women are faced with are absence of adequate care across the camps. Their opinions are presented in the Fig. 1.

Fig. 1 shows the major problems faced by Internally Displaced Persons across the sampled camps. Among the salient challenges identified in the course of the data collection were lack of adequate care, lack of freedom, financial problem, family disintegration and poor education. Lack of adequate care was mostly accounted for by respondents in Camps 1 and 3. This is due to their unmet expectation of being cared for in terms of good food, good shelter, proper clothing etc. by government, church authorities and other donors. Averagely, 20 of the elderly respondents representing 51.7 percent mostly reported this problem across the sampled camps, followed by 14 widowed (36.4 percent) and 14 married women (36 percent), while 10 single girls (26.1 percent) had the least responses. This suggests that the government and the management agencies seemingly need to improve the provision of healthcare, food, clothing, and shelter for IDPs in the selected camps.

The lack of freedom was also observed as a major problem observed by IDPs about the management of IDP camps. This was prominent among the single respondents in Camps 1 and 2 respectively. 11 single respondents averagely representing 28.2 percent in Camps 1 and 2 stressed lack of freedom as a major concern. This was followed by 3 widows and 3 elderly women with the equal percentage (7.5 percent) each while the married women had the least response of 2 (5 percent). The ability to move freely and in safety within and outside the camps is an important aspect for securing a sustainable livelihood. The findings suggest that regardless of the purpose of the move for tightened security by the government and relevant authorities, the conditions surrounding them are not supportive of their right to freedom of movement [10].

All the vulnerable categories of respondents except elderly women from Camp 1 and widows from Camp 3 emphasised the need for government and management agencies to provide modest financial supports that can help IDPs improve their economic power and finally help them reintegrate. The problem of finance is common among elderly respondents 6 (representing 15.8 percent) from Camps 2 and 3; followed by the married respondents 5 (representing 13.1 percent), 4 (10.4 percent) of the single girls and 4 (10.1 percent) of the widowed group. Majority of the responses 9 (23.7 percent) were derived from Camp 3. This coding theme had 10 percent responses from Camp 1. To improve finance and management of IDPs across the sampled areas, governments should solicit for donations and financial aids from humanitarian organizations and more international bodies across the globe. Platforms should be provided to ensure empowerment skills are translated into wealth.

Inadequate care for displaced children has also been observed as a problem in the IDPs camps. Most of the problems described at individual level were revealed at the family level as well, but in different magnitudes. The findings expressed the extent to which 5 married respondents (representing 13.2 percent) from Camp 2 were apprehensive about what the future holds for their children. While only 1 elderly respondent from the same camp (representing 2.6 percent) also supported the submissions of the married respondents. This level of apprehension can be associated with breakdown of familial and community systems, lack of basic and social amenities, and lack of schooling for their children. This coding theme had no supporting responses from Camps 1 and 2.

Family disintegration is one of the psychological and social challenges prevalent amongst some IDPs in the camps. Loss and deaths of family members still affects some IDPs. The effects of this is that they lack family ties as a coping mechanism in IDP camp. Only the widowed in Camp 2 agreed with the assertion that loss of family ties and family disintegration is a challenge to IDPs in camps. However, a large proportion of respondents stressed the prevalence of family disintegration as a challenge in Camp 3. On the average in camp 2, 2 married women (5.3 percent) having the highest percentage, while in camp 3, married women and the widowed having a representation of 2 responses each (5.3 percent) and only 1(2.6 percent) of the single girls supported the argument. This implies that strategic modalities should be placed on how to ensure the reintegration of families, enhance family ties and prevent future displacement.

The findings also revealed that response on social relations clashes in the sampled camps have slightly been on the increase especially amongst the single respondents in Camp 1. 1(2.5 percent) of girls in Camp 1 and 1 representation each of the single girls and the widowed group (representing 2.6 percent each) from Camp 2 supported social relation clashes as a challenge they face. Finally, the findings also indicated the level of education across the sampled camps is one of the challenges faced by the IDPs. Only 2 respondents of the married women group from Camp 1 (5 percent) and 2 respondents of the single girls group in Camp 3 representing (5.3 percent) alluded that displaced children lack access to schools and would grow up without an education if this is not addressed. This could definitely have national security concerns for Nigeria.

**Fig. 2:**
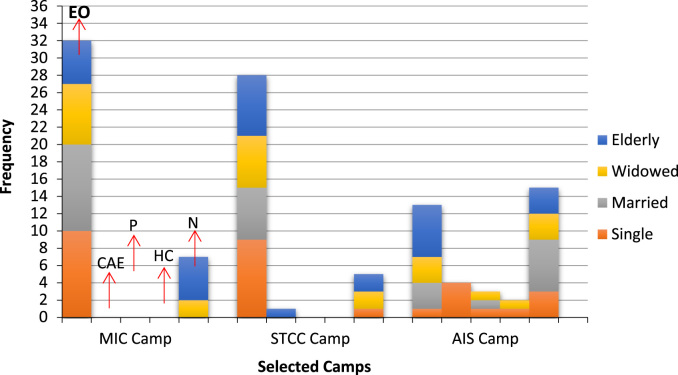
Coping mechanisms adopted by displaced women in the selected IDP camps. Keys: Economic Opportunities (EO); Children’s Access to Education (CAE); Prayer (P); Hoping for change (HC); Nothing (N).

After discussing the challenges faced by IDPs across the sampled IDPs, issues bothering on the coping mechanisms adopted amongst the displaced women were also discussed. Their responses from the various FGD sessions are presented below in Fig. 2.

Fig. 2 shows the coping mechanisms adopted by IDPs across the sampled camps. Majority of the respondents stressed the role of economic opportunities which comprised vocational services, skills acquisition and training in other traditional trades and skills, provision of financial assistance or support of income generating activities. In Camp 1, 10 single girls (25 percent) and 10 married women (25 percent) respondents took advantage of the economic opportunities followed by 7 of the widowed group (17.5 percent) and 5 elderly respondents (12.5 percent). In Camp 2, 9 of the single girls (23.7 percent) and 7 of the elderly women (18.4 percent) stressed the importance of economic opportunities. 6(15.8 percent) of the married women and widowed group respectively in Camp 2 also supported the assumption of the coding theme A (economic opportunities). In Camp 3, the elderly 6(15.8 percent), single girls 1(2.6%), married women3 (7.9%) as well as 3(7.9%) of the widowed participants of the FGD sessions use the benefits accrued from skills acquisition and vocational trainings as coping mechanisms for survival.

Education is critical to intellectual development and a major determinant for economic growth [7]. Education is also one of the major relief strategies for many displaced persons in camps. Only 4 the single girls’ category respondents representing 10.5 percent supported access to education as a coping mechanism in Camp 3. This is not so evident in Camps 1 and 2. In camp 2, elderly women had 1(2.6 percent) representation.

Prayer and religious fortitude were also important strategies for overcoming displacement distress. It was observed that only 1 (2.6 percent) each of the single girls, married women and widows categories of respondents in Camp 3 resorted to prayers and other forms of religious rituals as coping strategies. There was no support for this coding theme in camps 1 and 2. More so, only 3 of the married women category of respondents representing 7.9 percent see the combination of economic opportunities and access to education as coping mechanisms in Camp 2. This is not evident in Camps 1 and 3.

Additionally, family ties was also another way of coping with distress amongst displaced women. 1(2.5 percent) of the widowed group respondents from Camp 1, 1(2.6 percent) respondents of the married women group each from Camp 2 and another 1(2.6 percent) of the widowed respondents in Camp 3 attested to this claim of family ties being one of the coping strategies of IDPs. The result shows the importance of mutual support at the familial level, reinforcing the suitability of establishing community mobilization groups, and the creation of a peer-to-peer support system.

Other activities the respondents are engaged in to overcome the stress associated with displacement include group discussions amongst IDPs as well as recreational activities such as drought or cards games. The single girls and widowed respondents in Camp 3 often practice these activities. 1(2.6 percent) of IDPs in Camp 3 only coped by hoping for positive changes in their future. Furthermore, a larger percentage across the sampled IDPs camps did not think of anything to do to overcome the stress and negative feelings. Respondents in camps 1 and 2 did not support this coding theme. On the average 7(17.5 percent) of the respondents from Camp 1, 5(13.2 percent) of the respondents from Camp 2 and 15(39.5 percent) of the respondents from Camp 3 did nothing to cope with their situation of displacement. 1(2.6 percent) of the single girls and 1(2.6 percent) respondents in Camp 3 hoped for changes.

## 2 Experimental design, materials and methods

Four sessions of focus group discussions from each of the selected camps were conducted among participants. Respondents were categorized into single girls; married women; widowed and elderly women so as to capture the view of a cross section of the people on the issues of insurgency induced displacement and women’s coping strategies.

The FGD participants were referred to “A1 to A10”; “B1 to B10”; “C1 to C10” and “D1 to D10” for the groups. “A1 to A10” was used to represent the single girls groups; “B1 to B10” used to represent the elderly women groups; “C1 to C10” used to represent the widowed group and “D1 to D10 was used to represent the married women groups. These labels were replicated in referring to the four different FGDs groups across the different camps; they were replicated across the camps but were differentiated by the names of the camps in the body of the work where such responses were thematically presented.

Questions were linked directly to the objectives of the study. The advantage of using open-ended questions is that they do not steer or restrict the respondents’ answers. They gave room for flexibility of thoughts and gave opportunity to probe issues of interest [12]. Various techniques of probing informants were applied to encourage in-depth and insightful answers from the respondents. Questions for IDI were the same for all respondents except camp officials and women leaders that had different set of questions used to probe them. The FGDs were recorded with a tape recorder and were later transcribed into text.

The qualitative data collected from these separate FGD sessions were presented as texts, phrases and graphically to represent the experiences of the participants on the matters under review. Each session lasted between 105-150 minutes. These were subsequently transcribed, translated, codified and tabulated for consequent presentation graphically and statistically as deemed fit by the researcher. The final FGD texts was word processed for thematic analysis [4].

**Inclusion criteria:**

• Participants were Internally Displaced Persons of the sampled IDP camps;
• Participants signed the consent form provided and have stayed in the camp(s) for a minimum period of 2 years; and
• Participants were accessible at the time of the FGDs.

Data collected through focus-group discussions were audiotape-recorded and transcribed verbatim before analysis. Some of the data was also subjected to quantitative analysis. The method proposed by Elliot and Associates [2] was adopted for analysing the data generated and gathered. The model allowed for the data collected from the FGDs to be effectively managed and analysed.

**Analyse:**

i. After the entry of comments from respondents, the researcher searched for common themes that cut across the data obtained for each question and then enter them into excel sheets.
ii. After the consensus position is taken, the research thereafter assigned numbers and letters of the category that best fits each entry on the sheet.
iii. The research then grouped entries by the categories assigned to them.
  ■ When the researcher noticed that some of the entries were not consistent for their categories, the researcher re-categorised the information or added another category.
  ■ These processes were repeated for all the FGD sessions conducted on the basis of the research questions identified for the study.

In submission with the standards and other ethical principles on researches comprising human beings, attempt was made by the researcher to maintain the ethical principles with the intention of protecting the privacy and dignity of every individual that provided valuable information for the study. Request by informants to maintain animosity was obeyed. Finally, the identity of individuals from whom information was obtained in the course of the study was kept strictly confidential. Hence, names of FGD participants were coded to avoid identification by those who would have access to the publication of this research work, and information that could reveal the identity of the subject of the study was destroyed.
