# Supplementary material for: Datasets on the challenges of forced displacement and coping strategies among displaced women in selected Internally Displaced Persons׳ (IDPs) camps in Nigeria
Source: Data Brief. 2018 Jul 27;20:152–8. doi: 10.1016/j.dib.2018.07.042 (PMC6091441; doi:10.1016/j.dib.2018.07.042)
Supplement: Supplementary file 1 — Transparency document [file mmc1.docx]

**DECLARATION OF INTEREST FORM**

[**DATASETS ON THE CHALLENGES OF FORCED DISPLACEMENT AND COPING STRATEGIES OF DISPLACED WOMEN IN SELECTED INTERNALLY DISPLACED PERSONS’ (IDPs) CAMPS**](https://www.sciencedirect.com/science/article/pii/S2352340918302816) **IN NIGERIA**

**Olanrewaju, F. O.; Omotoso, F.;** **Alabi, J. O.**

Department of Political Science and International Relations, Covenant University, Ota, Nigeria

We, the Authors of paper entitled above certify that we have seen and approved the final version of the manuscript being submitted. This is an original work and has not received prior publication and is not under consideration for publication elsewhere. It is also important to state that there is no financial/personal interest or belief that could affect our objectivity and to prevent ambiguity, we humbly want to state explicitly that there is no conflicts of interest as regards the review and publication of this paper.

Thank you.

OLANREWAJU Faith Osasumwen

*Signed*
